# Supplementary material for: Evaluating sequence-to-function deep learning models for ancestry-stratified regulatory variant effect prediction using multi-ancestry blood eQTLs
Source: bioRxiv. 2026 Jun 26:2026.06.22.730889. Preprint. [Version 1] doi: 10.64898/2026.06.22.730889 (PMC13320881; doi:10.64898/2026.06.22.730889)
Supplement: Supplement 1 [file media-1.pdf]

Supplementary Information

Evaluating sequence-to-function deep learning models for ancestry-stratified regulatory variant effect prediction using multi-ancestry blood eQTLs

S1 Supplementary Figures

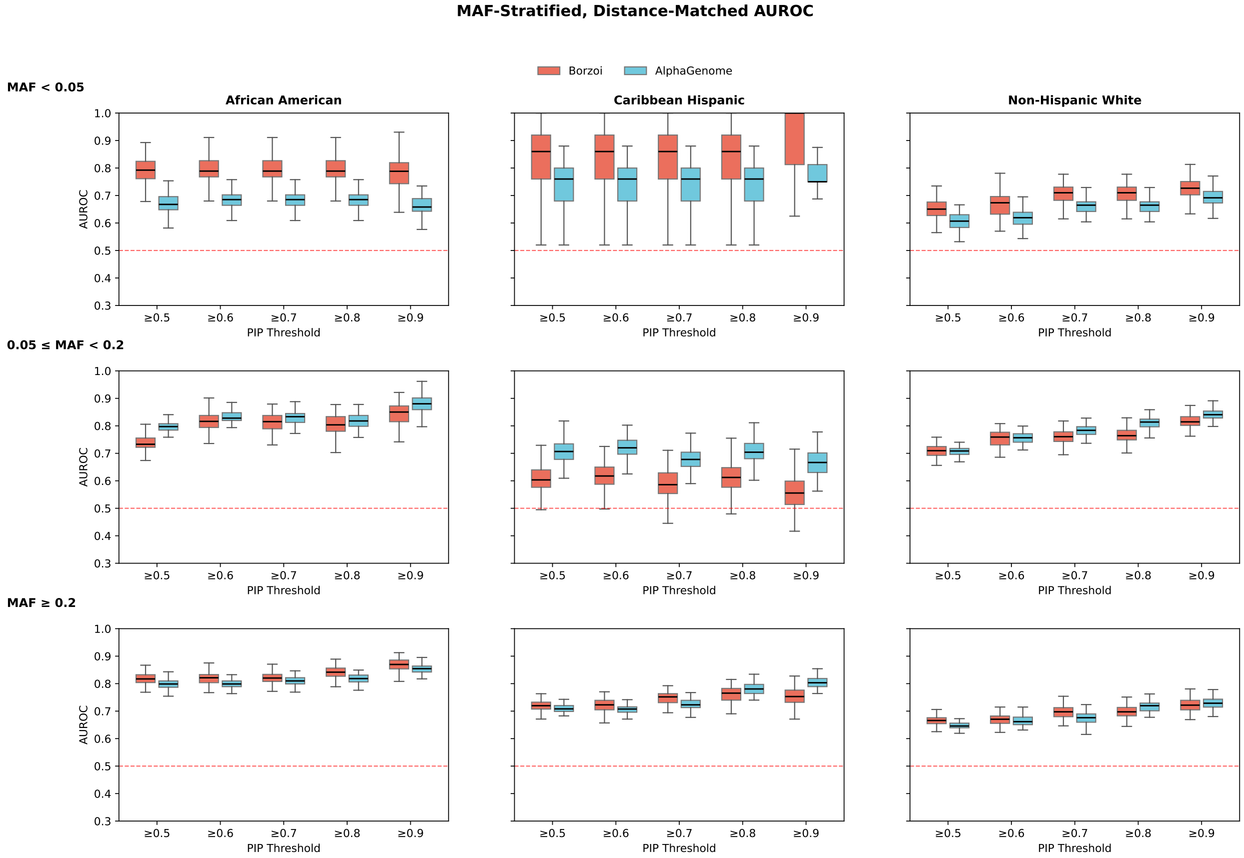

Figure S1: **MAF-stratified, distance-matched AUROC across ancestry groups and models.** AUROC was computed separately within each MAF stratum for African American (AA), Caribbean Hispanic (CH), and Non-Hispanic White (NHW) variants, using the same distance-matched framework as in the primary AUROC analysis. Columns denote ancestry groups and rows denote MAF strata. Within each panel, Borzoi and AlphaGenome are shown as side-by-side boxplots for each SuSiE PIP threshold (0.5–0.9), and each box summarizes 100 bootstrap iterations. The red dashed line indicates chance performance (AUROC = 0.5). Estimates in the MAF < 0.05 stratum, particularly for CH, should be interpreted cautiously because the number of positive variants is small.

S2 Supplementary Tables

Table S1: **Supplementary Table S1. Counts of scored fine-mapped eQTL–gene pairs by model, ancestry, PIP threshold, and TSS-distance bin for manuscript benchmark analyses.** Counts are shown for the deduplicated manuscript positive benchmark after restricting to positive pairs with a valid recomputed TSS distance and a model score. Rows with PIP $\geq$ 0.9 correspond to the high-confidence fine-mapped benchmark used in the fine-mapped panels of Figure 1 and the PIP $\geq$ 0.9 AUROC analyses in Figure 3. Inter-model convergence in Figure 2 uses the paired subset with scores from both Borzoi and AlphaGenome, so cell counts may be equal to or smaller than the per-model counts shown here. The n values shown in AUROC figure legends are smaller than these totals because positive and comparison pairs were subsequently distance-matched within log-distance bins before ROC calculation. Fine-mapped heatmap estimates based on $10 \leq n < 20$ are interpreted as exploratory; cells with $n < 10$ were omitted.

**Model**	**Group**	**PIP**	**Total**	**0–3 kb**	**3–12 kb**	**12–35 kb**	**>35 kb**
Borzoi	AA	$\geq$ 0.5	140	41 (29.3%)	25 (17.9%)	40 (28.6%)	34 (24.3%)
Borzoi	AA	$\geq$ 0.6	124	35 (28.2%)	24 (19.4%)	36 (29.0%)	29 (23.4%)
Borzoi	AA	$\geq$ 0.7	106	32 (30.2%)	21 (19.8%)	28 (26.4%)	25 (23.6%)
Borzoi	AA	$\geq$ 0.8	93	28 (30.1%)	18 (19.4%)	24 (25.8%)	23 (24.7%)
Borzoi	AA	$\geq$ 0.9	72	22 (30.6%)	13 (18.1%)	19 (26.4%)	18 (25.0%)
Borzoi	CH	$\geq$ 0.5	113	15 (13.3%)	28 (24.8%)	28 (24.8%)	42 (37.2%)
Borzoi	CH	$\geq$ 0.6	100	14 (14.0%)	23 (23.0%)	24 (24.0%)	39 (39.0%)
Borzoi	CH	$\geq$ 0.7	82	8 (9.8%)	20 (24.4%)	24 (29.3%)	30 (36.6%)
Borzoi	CH	$\geq$ 0.8	66	6 (9.1%)	18 (27.3%)	20 (30.3%)	22 (33.3%)
Borzoi	CH	$\geq$ 0.9	51	4 (7.8%)	14 (27.5%)	15 (29.4%)	18 (35.3%)
Borzoi	NHW	$\geq$ 0.5	313	75 (24.0%)	80 (25.6%)	68 (21.7%)	90 (28.8%)
Borzoi	NHW	$\geq$ 0.6	236	56 (23.7%)	58 (24.6%)	51 (21.6%)	71 (30.1%)
Borzoi	NHW	$\geq$ 0.7	184	48 (26.1%)	44 (23.9%)	38 (20.7%)	54 (29.3%)
Borzoi	NHW	$\geq$ 0.8	149	41 (27.5%)	36 (24.2%)	29 (19.5%)	43 (28.9%)
Borzoi	NHW	$\geq$ 0.9	117	31 (26.5%)	26 (22.2%)	24 (20.5%)	36 (30.8%)
AlphaGenome	AA	$\geq$ 0.5	147	41 (27.9%)	26 (17.7%)	41 (27.9%)	39 (26.5%)
AlphaGenome	AA	$\geq$ 0.6	131	35 (26.7%)	25 (19.1%)	37 (28.2%)	34 (26.0%)
AlphaGenome	AA	$\geq$ 0.7	113	32 (28.3%)	22 (19.5%)	29 (25.7%)	30 (26.5%)
AlphaGenome	AA	$\geq$ 0.8	100	28 (28.0%)	19 (19.0%)	25 (25.0%)	28 (28.0%)
AlphaGenome	AA	$\geq$ 0.9	79	22 (27.8%)	14 (17.7%)	20 (25.3%)	23 (29.1%)
AlphaGenome	CH	$\geq$ 0.5	113	15 (13.3%)	28 (24.8%)	28 (24.8%)	42 (37.2%)
AlphaGenome	CH	$\geq$ 0.6	100	14 (14.0%)	23 (23.0%)	24 (24.0%)	39 (39.0%)
AlphaGenome	CH	$\geq$ 0.7	82	8 (9.8%)	20 (24.4%)	24 (29.3%)	30 (36.6%)
AlphaGenome	CH	$\geq$ 0.8	66	6 (9.1%)	18 (27.3%)	20 (30.3%)	22 (33.3%)
AlphaGenome	CH	$\geq$ 0.9	51	4 (7.8%)	14 (27.5%)	15 (29.4%)	18 (35.3%)
AlphaGenome	NHW	$\geq$ 0.5	336	77 (22.9%)	83 (24.7%)	71 (21.1%)	105 (31.2%)
AlphaGenome	NHW	$\geq$ 0.6	255	58 (22.7%)	60 (23.5%)	53 (20.8%)	84 (32.9%)
AlphaGenome	NHW	$\geq$ 0.7	200	50 (25.0%)	46 (23.0%)	39 (19.5%)	65 (32.5%)
AlphaGenome	NHW	$\geq$ 0.8	165	43 (26.1%)	38 (23.0%)	30 (18.2%)	54 (32.7%)
AlphaGenome	NHW	$\geq$ 0.9	130	33 (25.4%)	28 (21.5%)	25 (19.2%)	44 (33.8%)

Table S2: **Supplementary Table S2. Positive-class MAF distributions across ancestries and PIP thresholds.** For each model, ancestry, and PIP threshold, the table reports the number of scored positive variants, the median minor allele frequency (MAF), and the percentage of positives with MAF < 0.05 for the deduplicated manuscript positive benchmark. These summaries show that AA positives are consistently shifted toward lower MAF than CH and are generally comparable to or lower than NHW, depending on model and threshold.

**Ancestry**	**PIP**	**Borzoi**			**AlphaGenome**		
		n	**Median MAF**	**MAF $<$ 0.05 (%)**	n	**Median MAF**	**MAF $<$ 0.05 (%)**
AA	≥ 0.5	140	0.229	12.1	147	0.228	12.9
AA	≥ 0.6	124	0.235	12.1	131	0.234	13.0
AA	≥ 0.7	106	0.239	14.2	113	0.237	15.0
AA	≥ 0.8	93	0.232	16.1	100	0.229	17.0
AA	≥ 0.9	72	0.220	16.7	79	0.214	17.7
CH	≥ 0.5	113	0.268	4.4	113	0.268	4.4
CH	≥ 0.6	100	0.268	5.0	100	0.268	5.0
CH	≥ 0.7	82	0.261	6.1	82	0.261	6.1
CH	≥ 0.8	66	0.251	7.6	66	0.251	7.6
CH	≥ 0.9	51	0.239	7.8	51	0.239	7.8
NHW	≥ 0.5	313	0.277	8.9	336	0.268	10.1
NHW	≥ 0.6	236	0.266	9.3	255	0.260	11.0
NHW	≥ 0.7	184	0.262	10.9	200	0.251	12.0
NHW	≥ 0.8	149	0.223	13.4	165	0.221	14.5
NHW	≥ 0.9	117	0.221	14.5	130	0.214	16.2

Table S3: **Supplementary Table S3. Summary of MAF-stratified, distance-matched AUROC across PIP thresholds.** Mean AUROC values are averaged across PIP thresholds 0.5–0.9 from the expanded 100× background analysis using available bootstrap estimates within each ancestry and MAF stratum. The n range gives the minimum and maximum number of positive variants contributing across those thresholds. Rare-variant estimates, especially for CH (n = 4–5), should be interpreted cautiously because of limited positive counts.

**Ancestry**	**MAF bin**	**Borzoi**		**AlphaGenome**	
		**Mean AUROC**	n **range**	**Mean AUROC**	n **range**
AA	MAF < 0.05	0.790	12–17	0.675	14–19
AA	$0.05 \leq$ MAF < 0.2	0.802	22–46	0.831	24–48
AA	MAF ≥ 0.2	0.833	38–77	0.816	41–80
CH	MAF < 0.05	0.851	4–5	0.748	4–5
CH	$0.05 \leq$ MAF < 0.2	0.596	12–24	0.694	12–24
CH	MAF ≥ 0.2	0.740	35–84	0.745	35–84
NHW	MAF < 0.05	0.692	17–28	0.647	21–34
NHW	$0.05 \leq$ MAF < 0.2	0.761	36–83	0.779	41–90
NHW	MAF ≥ 0.2	0.690	64–202	0.687	68–212

Table S4: **Supplementary Table S4. Sample sizes for FILER functional annotation overlap analysis.** Counts are deduplicated high-PIP positive variants available for each model, ancestry group, and PIP threshold.

**Model**	**PIP threshold**	**AA**	**CH**	**NHW**
AlphaGenome	≥ 0.5	142	104	325
AlphaGenome	≥ 0.6	126	93	245
AlphaGenome	≥ 0.7	108	75	193
AlphaGenome	≥ 0.8	96	60	158
AlphaGenome	≥ 0.9	76	46	123
Borzoi	≥ 0.5	136	104	309
Borzoi	≥ 0.6	120	93	233
Borzoi	≥ 0.7	102	75	183
Borzoi	≥ 0.8	90	60	148
Borzoi	≥ 0.9	70	46	116

Table S5: **Supplementary Table S5. Key FILER functional annotation overlap among high-PIP variants.** Values are percentages of high-PIP positive variants overlapping selected FILER categories emphasized in the manuscript. Chi-square q values are Benjamini–Hochberg FDR-adjusted 3×2 tests comparing AA, CH, and NHW within each model and PIP threshold. For readability, this compact table reports the least and most stringent PIP thresholds.

Model	PIP	Category	AA (%)	CH (%)	NHW (%)	Chi-square q
AlphaGenome	≥ 0.5	Accessible chromatin	70.4	63.5	54.8	0.021
AlphaGenome	≥ 0.5	Hi-C	76.8	74.0	63.1	0.021
AlphaGenome	≥ 0.5	IM-PET	48.6	43.3	34.5	0.034
AlphaGenome	≥ 0.5	pcHi-C	90.1	90.4	85.8	0.327
AlphaGenome	≥ 0.5	eQTL	83.1	96.2	95.1	1.86e-04
AlphaGenome	≥ 0.5	sQTL	48.6	73.1	62.5	0.003
AlphaGenome	≥ 0.9	Accessible chromatin	78.9	67.4	65.9	0.393
AlphaGenome	≥ 0.9	Hi-C	76.3	65.2	59.3	0.186
AlphaGenome	≥ 0.9	IM-PET	52.6	47.8	43.1	0.573
AlphaGenome	≥ 0.9	pcHi-C	92.1	84.8	78.0	0.163
AlphaGenome	≥ 0.9	eQTL	81.6	97.8	93.5	0.053
AlphaGenome	≥ 0.9	sQTL	50.0	65.2	60.2	0.429
Borzoi	≥ 0.5	Accessible chromatin	69.9	63.5	55.7	0.056
Borzoi	≥ 0.5	Hi-C	77.2	74.0	63.4	0.034
Borzoi	≥ 0.5	IM-PET	49.3	43.3	35.6	0.062
Borzoi	≥ 0.5	pcHi-C	90.4	90.4	87.7	0.647
Borzoi	≥ 0.5	eQTL	84.6	96.2	96.8	5.75e-05
Borzoi	≥ 0.5	sQTL	49.3	73.1	63.1	0.005
Borzoi	≥ 0.9	Accessible chromatin	78.6	67.4	69.0	0.468
Borzoi	≥ 0.9	Hi-C	77.1	65.2	60.3	0.242
Borzoi	≥ 0.9	IM-PET	54.3	47.8	45.7	0.709
Borzoi	≥ 0.9	pcHi-C	92.9	84.8	80.2	0.242
Borzoi	≥ 0.9	eQTL	84.3	97.8	95.7	0.075
Borzoi	≥ 0.9	sQTL	51.4	65.2	61.2	0.468

Table S6: **Supplementary Table S6. Selected pairwise ancestry comparisons for key FILER annotation categories.** Rows show pairwise Fisher exact tests with FDR-adjusted $q < 0.10$ for the compact set of categories discussed in the manuscript at PIP thresholds shown in Supplementary Table S5. Fractions are percentages of high-PIP variants overlapping each category; difference is ancestry 1 minus ancestry 2 in percentage points.

Model	PIP	Category	Comparison	Frac. 1 (%)	Frac. 2 (%)	Diff.	q
AlphaGenome	≥ 0.5	Accessible chromatin	AA vs NHW	70.4	54.8	+15.7	0.017
Borzoi	≥ 0.5	Accessible chromatin	AA vs NHW	69.9	55.7	+14.2	0.050
AlphaGenome	≥ 0.5	Hi-C	AA vs NHW	76.8	63.1	+13.7	0.034
Borzoi	≥ 0.5	Hi-C	AA vs NHW	77.2	63.4	+13.8	0.050
AlphaGenome	≥ 0.5	IM-PET	AA vs NHW	48.6	34.5	+14.1	0.038
Borzoi	≥ 0.5	IM-PET	AA vs NHW	49.3	35.6	+13.7	0.054
AlphaGenome	≥ 0.5	eQTL	AA vs CH	83.1	96.2	-13.1	0.016
AlphaGenome	≥ 0.5	eQTL	AA vs NHW	83.1	95.1	-12.0	0.002
Borzoi	≥ 0.5	eQTL	AA vs CH	84.6	96.2	-11.6	0.050
Borzoi	≥ 0.5	eQTL	AA vs NHW	84.6	96.8	-12.2	8.17e-04
AlphaGenome	≥ 0.5	sQTL	AA vs CH	48.6	73.1	-24.5	0.003
AlphaGenome	≥ 0.5	sQTL	AA vs NHW	48.6	62.5	-13.9	0.038
Borzoi	≥ 0.5	sQTL	AA vs CH	49.3	73.1	-23.8	0.005
Borzoi	≥ 0.5	sQTL	AA vs NHW	49.3	63.1	-13.8	0.050
